# A Systematic Review on the Use of Registration-Based Change Tracking Methods in Longitudinal Radiological Images

**DOI:** 10.1007/s10278-024-01333-1

**Published:** 2024-11-22

**Authors:** Jeeho E. Im, Muhammed Khalifa, Adriana V. Gregory, Bradley J. Erickson, Timothy L. Kline

**Affiliations:** Department of Radiology, Mayo Clinic, 200 First St SW, Rochester, MN 55905 USA

**Keywords:** Image registration, Longitudinal, Change tracking, Machine learning, Subtraction, Deformable registration

## Abstract

Registration is the process of spatially and/or temporally aligning different images. It is a critical tool that can facilitate the automatic tracking of pathological changes detected in radiological images and align images captured by different imaging systems and/or those acquired using different acquisition parameters. The longitudinal analysis of clinical changes has a significant role in helping clinicians evaluate disease progression and determine the most suitable course of treatment for patients. This study provides a comprehensive review of the role registration-based approaches play in automated change tracking in radiological imaging and explores the three types of registration approaches which include rigid, affine, and nonrigid registration, as well as methods of detecting and quantifying changes in registered longitudinal images: the intensity-based approach and the deformation-based approach. After providing an overview and background, we highlight the clinical applications of these methods, specifically focusing on computed tomography (CT) and magnetic resonance imaging (MRI) in tumors and multiple sclerosis (MS), two of the most heavily studied areas in automated change tracking. We conclude with a discussion and recommendation for future directions.

## Introduction

The tracking and evaluation of biological changes is an important clinical task. Many diseases have a variety of presentations and progressions. For instance, multiple sclerosis (MS) lesions may grow, shrink, form, or disappear over time [1]. In addition, it is important to track how diseases respond to different treatments. In the case of cancer, tumors may grow or shrink depending on the type and dose of treatment provided [2]. Therefore, the longitudinal analysis of clinical changes can help a clinician evaluate each patient and decide on the best course of treatment.

Radiological images like computed tomography (CT), magnetic resonance imaging (MRI), X-rays, and ultrasound make the longitudinal tracking of disease progression possible. Images taken at different time points can be directly compared with each other to evaluate longitudinal changes. However, one problem is that successive images may differ in modality, patient orientation, position, or anatomy, complicating the analysis. To account for these differences, registration is used to align the images. Image registration is the process of aligning images taken under different conditions and/or at different times [3]. Such transformations and alignments allow for direct comparison of certain features, like cysts, tumors, and organs, in longitudinal images.

The first goal of this review is to provide an overview of the different types of registration methods, and how registration can facilitate change tracking in radiological imaging. The second part of this article explores the two main methods of detecting and computing change once the images are registered: the intensity-based approach and the deformation-based approach. Lastly, the authors investigated the clinical applications of these methods in cancer and MS, two of the most heavily studied areas in automated change detection and tracking.

### Registration

The goal of registration is to align images taken under different conditions by transforming the *moving image* to spatially and temporally match the *fixed image* [4]. Registration algorithms use correspondences between images like landmarks, features, or intensities to guide the transformation of the images [5]. Landmark-based registration requires the user to select anatomical landmarks common to each image based on prior knowledge. The algorithm then estimates the transformations between the images based on these markers [5, 6]. Feature-based registration identifies various features, like color, gradient, edges, shape, and surface contour, consistent in both images to estimate the transformations. Intensity-based registration uses similarity cost functions to compute how similar the intensity values are between images at the voxel level. Registration is optimized until the cost function between the images is minimized and the similarity is maximized [6–8]. The three most used cost functions are the sum of square difference (SSD), cross-correlation (CC), or mutual information (MI). SSD is one of the most basic cost functions and involves subtracting each voxel between two images individually and squaring and summing the difference. SSD requires the two images to have the same intensity range, which may make it more susceptible to errors due to artifacts and noise. CC finds the degree of similarity between two images by vectorizing the image. This function is intensity normalized, making it robust to intensity shifts between successive images. MI involves using a probabilistic cost function to find similarity measures, making it more robust to noise and intensity shifts. Unlike other functions, MI compares how intensities between images are interdependent rather than directly comparing intensity values, making it more suitable for registering different image modalities [4]. Although intensity-based methods do not require user input, a user will often align the images manually before applying the registration method [5].

**Fig. 1:**
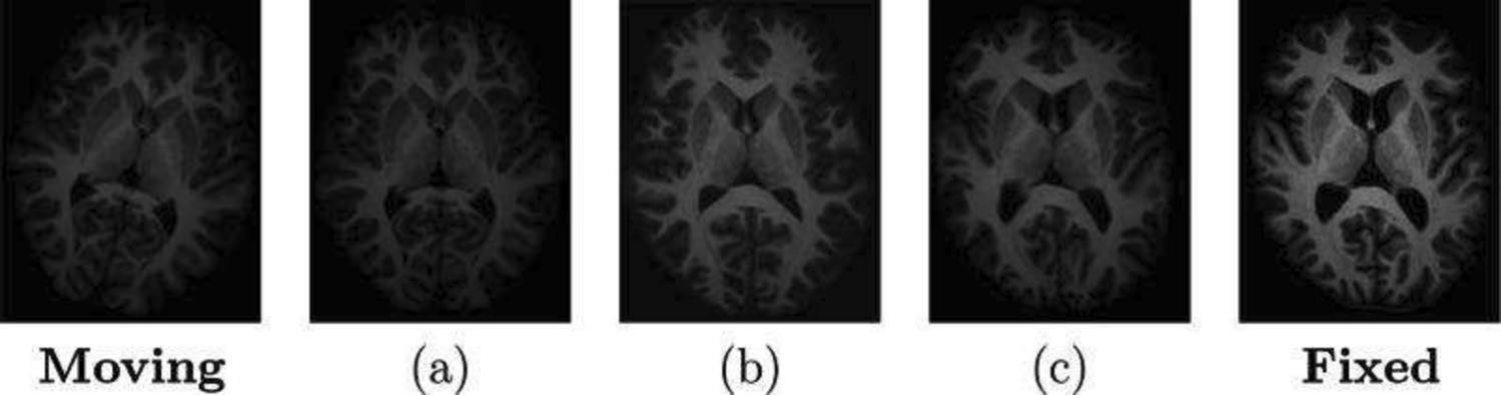
MRI images of the brain demonstrating the results of using three registration types between a moving and fixed image. Rigid registration was used in **a**, affine in **b**, and nonrigid/deformable registration in **c**. Image retrieved and modified from Chen et al. [4], 2024 Springer Nature

There are three main registration types: rigid, affine, and nonrigid. The type of registration used depends on the desired degrees of freedom and modality of the images. Rigid registration is applicable when significant transformations are not required. In such cases, a global transformation, like translations and rotations, is sufficient to align the images [4]. For instance, in images of the heart taken at the same time of a cardiac cycle in the same patient, little transformation is expected, and rigid registration is sufficient to align the images [7]. Affine registration is an extension of rigid registration which allows for additional shearing and scaling [9]. Affine registration is most suitable in cases where single rigid registration would not sufficiently align the images and nonrigid registration would inaccurately distort image features [10]. On the other hand, nonrigid (or deformable) registration is used when there are significant differences between the images, and more complex transformations are required. In nonrigid registration, transformations are performed locally at the voxel level using displacement fields [4]. This process is also called deformable registration because the moving image is spatially deformed to match the fixed image. Nonrigid transformations are often preferable in clinical applications as they allow for the registration of images from different modalities and orientations or those taken at different time points. However, due to its high degree of freedom, this method is difficult to implement with high accuracy and speed [11]. Therefore, in clinical applications, nonrigid registration is necessarily an automated process and is often preceded by rigid and/or affine registration to minimize error and enhance performance [12]. Figure 1 shows the three types of registration performed on MR images of the brain. In practice, nonrigid approaches will compute a cascade of transformations going from rigid → affine → nonrigid.

### Automated Methods of Registration

Manual registration is possible but is not ideal due to poor reproducibility, user bias, and lack of a metric or loss function to measure its performance [5]. Therefore, most registration methods are either automated or semi-automated. Traditional automated registration methods use algorithms to find the displacement vector of each voxel between the moving and fixed image. Based on the resulting displacement vector field, the moving image is warped to match the fixed image, and the transformations are optimized iteratively using a similarity metric until the convergence of the images is satisfactory [13, 14]. As this process is iterative and requires complex computations, the transformations take a long time to be computed.

Therefore, more recently, deep learning-based registration methods have been explored. Initially, deep learning was used to detect misalignment and increase accuracy in registration. Now, deep learning models can learn a transformation function from a sufficiently large dataset of moving and fixed images and apply it to perform registration accurately and rapidly [13, 15]. The advantage of deep learning-based algorithms is that after they have been trained, they can then be applied to perform registration non-iteratively and rapidly without the need for complex optimization computations for each case. Unlike traditional methods, they also require minimal pre-processing of the images and minimize user bias [16]. The three most common techniques for deep learning-based registration are convolutional neural networks (CNN), reinforcement learning (RL), and generative adversarial networks (GAN) [17–20].

The training of deep learning-based registration models can largely be supervised or unsupervised. Supervised registration models are trained on a large input training set of moving and fixed images, as well as corresponding reference standards, registered images, and/or transformations [4, 13]. One downside to supervised methods is that these reference standards may be time-consuming and difficult to acquire. Employing unsupervised models can help resolve this issue as these models only require the moving and fixed images as the input. Instead of learning the transformations from a reference standard, the unsupervised model is optimized with a loss function to minimize the dissimilarity between the fixed image and the warped moving image [3, 4, 13]. Although these deep learning-based registration models have tremendous potential, their performance is still not ideal in all circumstances. Therefore, it is crucial to design the appropriate registration model based on the available dataset and desired degrees of freedom [5, 14].

### Automated Methods of Change Tracking

Once pairs of longitudinal images are registered, comparisons of various features (e.g., organs, cysts, tumors, lesions) are possible. Previous studies [21–23] have used non-registration-based methods to track longitudinal changes in images, like conducting volumetric analysis of lesions in non-registered longitudinal images. However, these methods rely entirely on manual segmentations of the feature of interest, which may be time-consuming and highly variable across users, and do not account for differences in patient position, anatomy, or image modalities. Comparing features using this method is even more difficult when multiple cysts or lesions need to be evaluated individually.

**Fig. 2:**
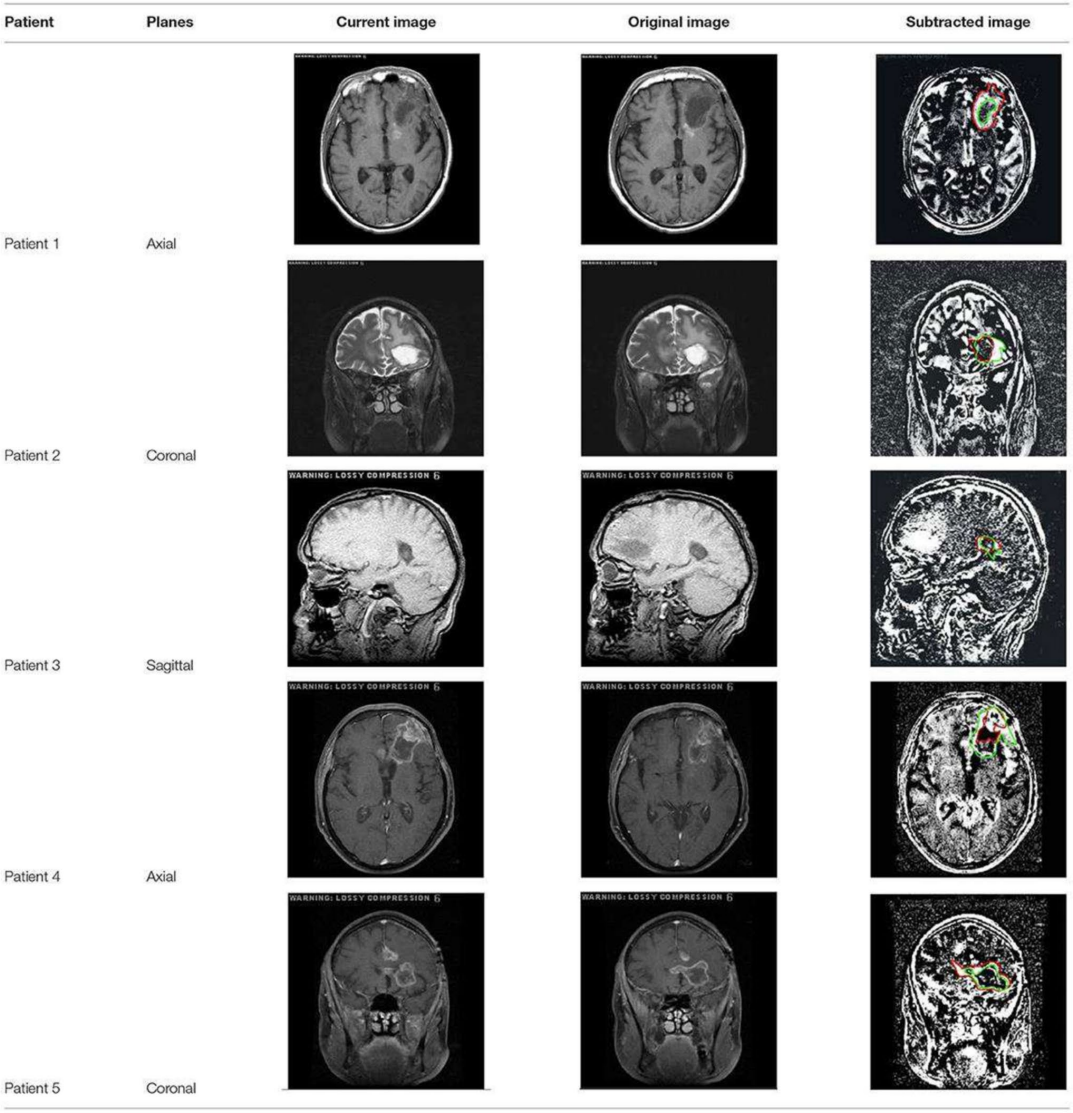
A depiction of five different results of the subtraction technique between previous (original) and current images at two different time points in five patients with brain tumors. In patient 1, the subtraction image demonstrates a regression in tumor size. Subtraction images in patients 2 and 4 exhibit progression/enlargement in tumor size. Subtracted images in patients 3 and 5 showed a difference in lesion location. Image retrieved and modified from Khalil et al. [29], 2024 Frontiers Media

The two major ways that information from registration can be used to detect and quantify changes include the intensity-based approach and the deformation-based approach [2, 24, 25]. We mentioned above in the “Registration” section the different types of registration (rigid vs affine vs non-rigid, or landmark-based vs feature-based vs intensity-based registration), and after registration is complete, we can quantify the changes using approaches such as intensity-based approach (e.g., subtraction techniques) and deformation-based approach (e.g., deformation field analysis). In the intensity-based approach, comparisons are made at the voxel level. For instance, subtraction methods can be used to essentially subtract intensity values at the voxel level between the registered images to produce subtraction images that both visually and computationally represent the changes [26–31]. Therefore, intensity-based comparison is most suitable for detecting new or disappearing lesions. Figure 2 illustrates an example of the subtraction method [29].

The only downside to the subtraction method is that it only considers individual voxel-wise changes and it is also susceptible to noise. Therefore, machine learning-based methods, such as probabilistic classification algorithms, can automatically classify voxel-wise and lesion-wise changes between registered images [32, 33]. Unlike the subtraction method, probabilistic classification algorithms can help incorporate shape and contextual information to automatically generate a segmentation map of new or enlarged lesions [32, 34–36]. Deep learning-based models have also been developed to produce masks of lesion changes based on an input of baseline (BL) and follow-up (FU) images [1, 16, 20, 25, 37, 38].

**Fig. 3:**
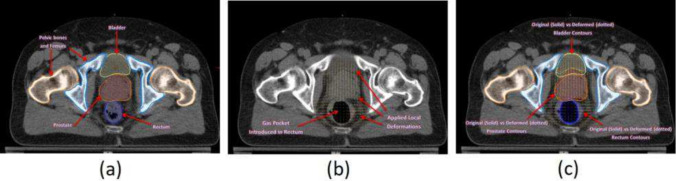
Axial view of a prostate CT image with notable organs (pelvis, bladder, rectum, prostate) segmented. The figure illustrates how the analysis of deformation fields can facilitate the tracking of anatomic changes. **a** shows the segmentation of organs in the initial CT image, **b** shows the deformation fields of local changes induced by a gas pocket in the rectum, and **c** shows the segmentation of the changed structures of these organs, compared to the original segmentation. Image retrieved and modified from Varadhan, R. et al. [43], 1999–2024 John Wiley, Inc or related companies

In the deformation-based approach, the displacement vector field acquired from deformable registration can be analyzed directly to quantify the changes. As displacement vectors indicate differences in images, a divergence operator can compute structural and volumetric changes from the vector field [39, 40]. Deformation-based comparison is most applicable for detecting shrinking or enlarging of existing lesions. Deep learning-based methods have also been used to analyze deformation fields (DF) to detect change without the need for user input [41]. More recently, deep learning-based methods have combined the two approaches to train models on subtraction images and deformation field operators to detect longitudinal lesion change more accurately [8, 27, 42]. Figure 3 shows an example of deformation field analysis to track structural changes.

## Methods

We searched for eligible articles in PubMed and Google Scholar until March 2024. The following keywords and subject headlines were used in the search strategy: “registration” “rigid” “tracking” “longitudinal” “machine learning” “deep learning” “deformable” “MS” “tumor” “cancer” and “subtraction. Only 161 articles were selected to be reviewed from the databases. The search was restricted to only human subjects and the English language. Of the 161 articles, 69 were included and 92 were excluded based on our inclusion/exclusion criteria.

### Inclusion/Exclusion Criteria

The authors focused on articles concerning registration-based methods for tracking changes in longitudinal images, including both the traditional and deep learning-based approaches. For the clinical applications, only articles about tumors and multiple sclerosis and only articles using MRI or CT scans were included.

**Fig. 4:**
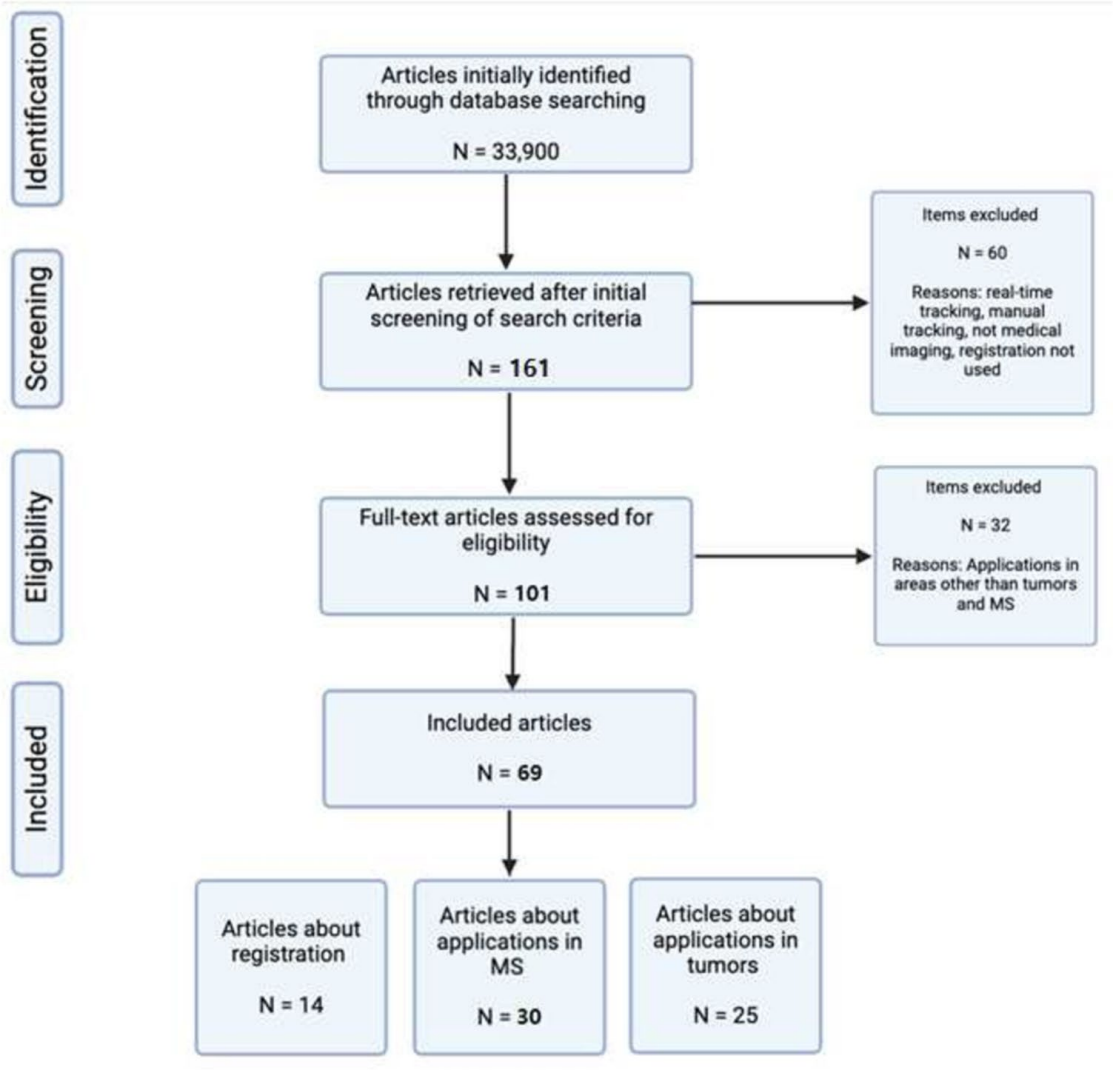
Summary flowchart of included and excluded

Articles that did not use registration-based methods for tracking changes were not included. Also, articles that explored real-time change tracking during surgery or radiotherapy, were not within the scope of this article and were also excluded. We focused exclusively on longitudinal images to provide a comprehensive analysis of the role of registration in this specific area. Because we noticed that multiple reviews have addressed the real-time aspect, but there is a notable scarcity of literature focusing on longitudinal images. Abstracts not accompanied by full text were also not included (refer to Fig. 4 for a summary flowchart of the inclusion/exclusion criteria).

## Applications in Multiple Sclerosis

Change detection in multiple sclerosis (MS) is crucial because of the progressive nature of the disease. Tracking how MS white matter lesions change over time can be vital to assessing disease progression and treatment response [33]. The authors investigated 30 studies that explored registration-based methods to track changes in MS lesions. All studies of MS used magnetic resonance imaging (MRI). Out of the 30 studies, rigid or affine registration was used in 16 articles, and nonrigid registration was used in 10. Most articles that used nonrigid registration started with rigid alignment as the first step. In 4 of the articles, the registration type was not specified. Methods used to quantify the changes after registration varied greatly between studies.

Twenty-three articles used the intensity-based approach to track longitudinal changes after registration. The most common method, image subtraction, was included in 14 studies [8, 26, 27, 31, 42, 44–52] and was mostly preceded by rigid or affine registration. The main clinical advantage of subtraction images was that they served as a reference for clinicians to detect lesion changes. For instance, in a study of 40 MS patients with active lesions, Moraal, B., et al. [48] showed a 1.7-fold increase in the detection of positive active lesion changes and a 2.9-fold increase in the detection of negative lesion changes when the reader used subtraction images as a reference. While previous studies used 2D magnetic resonance (MR) images for subtraction, Moraal, B., et al. [47] were among the first to use three-dimensional (3D) subtraction imaging to compare successive MR images of 14 active MS patients. 3D subtraction allowed for small changes to be identified, which increased lesion detection accuracy and minimized noise due to flow or misregistration. Tan, I. L., et al. [45] similarly showed a 35% increase in the detection of positive active lesion change when using 3D FLAIR images for subtraction compared to 2D MRIs. Several studies used deep learning-based image subtraction. Battaglini, M., et al. and Ganiler, O., et al. [26, 27] reported fully automated, unsupervised pipelines to generate subtraction images for longitudinal MR images of MS patients with respective sensitivities of 0.91 and 0.85. Compared to manual methods, the fully automated subtraction pipelines showed a reduction in the number of false positives and an increase in sensitivity. These studies show the potential of the subtraction method for change tracking/evaluation, as they are time- and cost-efficient and sensitive to small changes.

As subtraction methods only compare images at the voxel level, some studies further improved intensity-based change detection methods with probabilistic classification algorithms [32, 35, 53]. Unlike previous methods, these methods allowed for the classification of change at the lesion level (as opposed to the voxel level), which enables the incorporation of contextual information around the lesions. This allowed for better differentiation between intensity differences due to lesion changes and those due to artifact noise or misregistration. For instance, in a clinical trial of 160 MS patients, Elliott, C., et al. [32] employed a Bayesian classifier to identify new lesion candidates between successive MR scans and a random-forest classifier to classify these candidates as true lesions within the context of various features like neighboring voxels, tissue type, lesion size, and noise artifacts. The classifiers produced segmentation maps of lesion changes with a sensitivity of 0.99 and a false detection rate of 0.02 when assessing new lesions greater than 0.15 cc in size. Similarly, Cheng, M., et al. [53] used texture descriptors to further classify lesion change candidates acquired from image subtraction and exhibited improved change detection when compared to previous methods that only subtracted images at the voxel level [34]. Since radiologists typically annotate lesions based on the larger context of the surrounding regions rather than individual voxels, incorporating contextual and texture information is likely more informative in a clinical setting.

Other studies [1, 16, 25, 37, 38, 52, 54, 55] used deep learning-based methods. Krüger, J., et al. [37] trained a supervised, convolutional neural network (CNN)-based model to output a 3D mask of new or enlarged lesions given an input of BL and FU fluid-attenuated inversion recovery (FLAIR) images. The model improved previous methods by considering both BL and FU images simultaneously (rather than individually) to produce segmentations of the lesion changes. Compared to traditional manual segmentation methods, like the Lesion Segmentation Toolbox (LST), the average computation time decreased from 30 to 1 min [37]. Whereas previously supervised models [37] required a large, annotated training set, To, M.-S., et al. [16] employed an unsupervised Siamese 3-D model, which did not require an annotated training set, based on focal Tversky loss to generate segmentation maps of lesion changes automatically. Given the diverse ways in which lesion changes can occur, annotating every possible type of change in the training set would be impractical. The unsupervised method does not require a reference standard. Thus, it could be more suitable in cases where the type of change cannot be anticipated. Also, the lack of need to manually annotate a training set saved time in the data preparation step. Uwaeze, J. et al. [52] similarly developed unsupervised models called locally linearly embedding (LLE) and isometric feature mapping (Isomap) using subtraction images as the ground truth. These models accurately produced segmentations of active MS lesions, and when compared to the ground truth, outperformed state-of-the-art methods for lesion detection. The models also showed promise in identifying active lesions in non-contrast brain MRI, suggesting they could reduce the need for administration of gadolinium-based contrast agents and thus reduce patient risk and healthcare costs. Compared to traditional methods, the advantage of deep learning-based methods is they can improve performance in terms of both accuracy and speed. Also, these models are better able to resemble the performance of radiologists by capturing complex relationships between variables in the images [16, 37]. The group employed an unsupervised Siamese 3-D model based on focal Tversky loss to generate segmentation maps of lesion changes automatically. Because lesion changes may occur in various ways, annotating every type of change in the training set would not be feasible. Because the unsupervised method does not require a reference standard, it could be more suitable in cases where the type of change cannot be anticipated.

In total, 5 studies [39–42, 56] used the deformation-based approach to detect changes. Rey, D., et al. [39] analyzed deformation fields to produce segmentations of local lesion changes and employed a Jacobian operator—a vector displacement field operator—to quantify local volumetric changes in the displacement vector field. Cabezas, M., et al. [42] further expanded on this method by combining DF analysis with the subtraction method. Following up the subtraction method with DF analysis significantly decreased false positives, compared to traditional subtraction methods, and resulted in a Dice coefficient of 0.79 for detecting new lesions and a Dice coefficient of 0.60 for segmentation and volumetric calculations. Salem, M., et al. [41] used an unsupervised, fully convolutional neural network-based approach to combine the intensity-based and deformation-based methods. The model first learned the DFs and the nonrigid registration of the images and then learned to produce a segmentation map of new T2-w lesions based on the DFs. The model achieved a Dice coefficient of 0.83 and a true positive detection rate of 83.09%. The model also performed faster and with higher accuracy in detecting new lesions than previous methods of DF analysis [8, 42] (refer to Table 1 for a summary of the results).

**Table 1:** Summarizes the results of the articles related to the clinical application of registration-based change tracking in MS including the type of registration and the method used to detect changes. We selected multiple examples of each method used to detect changes

First Author	Year	Type of registration	Change tracking method	Pathology	Summary of results
Moraal, B., et al	2009	Affine	Subtraction images	MS	When the expert reader used subtraction images for reference, they showed a 1.7-fold increase in the detection of positive active lesion changes and a 2.9-fold increase in the detection of negative lesion changes
Ganiler, O., et al	2014	Rigid	Subtraction images	MS	A fully automated, unsupervised subtraction pipeline, which incorporated tissue classification, helped detect white matter MS lesion changes
Jain, S., et al	2016	Rigid and affine	Subtraction images	MS	An unsupervised joint expectation maximization framework produced segmentations of evolving lesions based on image subtraction
Sepahvand	2020	Unspecified	Subtraction images	MS	A 3D Unet segmentation segmentation network trained using subtraction images accurately detected and segmented MS lesions in both BL and FU images
Adoum, A., et al	2023	Rigid	Subtraction images	MS	A co-registration subtraction with the lesion color-coding model significantly increased detection of new MS lesions and decreased mean reading time
Uwaeze, J., et al	2024	Unspecified	Subtraction images	MS	Unsupervised models using subtraction images as ground truth accurately produced segmentations of active MS lesions in noncontrast MRI
Bosc, M., et al	2003	Affine and nonrigid	Deep learning model	MS	After subtraction, a generalized likelihood ratio test helped detect significant lesion changes while eliminating artifactual changes
Elliott, C., et al	2013	Affine and nonrigid	Deep learning model	MS	Bayesian and random forest classifiers helped identify lesion change candidates and classify them as real change. The classifiers produced segmentation maps of lesion changes
To, M., et al	2021	Rigid	Deep learning model	MS	An unsupervised Siamese 3-D model based on focal Tversky loss automatically generated segmentation maps of lesion changes
Calvi, A., et al	2023	Nonrigid	Deformation analysis	MS	A convolutional neural network using deformation analysis computationally assessed the evolution of incident MS lesions into slowly expanding lesions
Raj, A., et al	2024	Rigid	Deformation analysis	MS	Voxel-guided morphometry was used to extract volume alterations of MS lesions from the deformation field
Cabezas, M., et al	2016	Nonrigid	Combine deformation analysis and subtraction	MS	Combining deformation analysis with the subtraction method improved the performance of detecting new lesions and calculating volumetric changes, compared to traditional subtraction methods
Salem,M., et al	2018	Affine and nonrigid	Combine deformation analysis and subtraction	MS	An unsupervised, fully convolutional neural network-based approach that combined the subtraction and deformation methods resulted in faster and more accurate performance in producing segmentations of lesion changes compared to previous DF analysis methods
Salem, M., et al	2020	Nonrigid	Combine deformation analysis and subtraction	MS	A supervised framework helped detect new T2-w lesions using information from image intensities, subtraction values, and deformation fields

## Applications in Tumors

Registration has a critical role in oncology, especially in radiation oncology. Registration can be used to help clinicians in a variety of ways. One benefit of registration is facilitating image-guided therapy during surgery or radiotherapy (not included in the search criteria). Another important advantage of registration is to help quantify responses from tumor treatments [57]. Cancerous lesions can follow different paths over time when following patients with serial scans. They can shrink or remain stable, or new lesions may be formed even with treatment. Detecting changes in lesions, especially in the early stages is crucial to planning an effective course of treatment. Currently, RECIST (response evaluation criteria in solid tumors) is the guideline for evaluating tumor progression and treatment efficacy. RECIST classifies each tumor progression into four categories: complete response (complete disappearance of tumor), partial response (reduction in tumor size), partial progression (increase in tumor size), and progressive disease (no significant change). However, this diagnostic tool has shown a relatively poor association with clinical outcomes because it is unable to compute volumetric changes of tumors and overlooks many metastatic tumors [58]. Therefore, registration-based methods are utilized to overcome such problems and match the lesions to help monitor changes over time.

We included 25 articles that used registration-based methods to track changes in longitudinal images of tumors. Rigid or affine registration was used in 5 studies, and nonrigid registration was used in 16 studies. Most articles that used nonrigid registration started with rigid alignment as the first step. In 4 of the articles, the registration type was not specified.

In total, 14 articles used the intensity-based approach to track longitudinal changes after registration, and 11 articles [29, 30, 59–67] used subtraction methods to compare tumors after registration. Staring, M., et al. [59] non-rigidly registered longitudinal chest CT images of 33 individuals who had focal ground-glass opacities during lung cancer screening. Then, subtraction images were acquired to aid observers in detecting malignant tumors by showing increases in volume or density. When using subtraction images for reference, agreement with the standard of reference increased from 0.61 to 0.76 for volume change and from 0.53 to 0.64 for density change, which indicates improvement in change detection. Similarly, Sakamoto, R., et al. [60] performed nonrigid registration and temporal subtraction to improve radiologist performance in detecting bone metastases in CT images. The subtraction images enhanced the detection of small lesions and significantly reduced reading time for radiologists. Nakaura, T., et al. [30] employed rigid registration and subtraction to detect focal enhancement in hypervascular hepatocellular carcinoma patients. They found that when using subtraction images, the mean area under the best-fit ROC curve values for observer performance increased from 0.86 to 0.91.

Other studies [28, 68–74] used deep learning-based methods to compare intensity values between successive images. For instance, Rudie, J. D., et al. [68] trained a 3D U-Net neural network on 397 registered and subtracted FLAIR and T1 images to localize and quantify glioma changes posttreatment. The model performed with an accuracy of 90–91% when classifying longitudinal changes in active tumors. A separate CNN model was also trained to perform segmentations of posttreatment gliomas for longitudinal volumetric analysis. Other studies [2, 28, 69–71] used deep learning to improve the automatic segmentation of tumors and automatically analyze volumetric changes. Vandewinckele, L., et al. and Hering, A., et al. [69, 70] found an improvement in the automatic segmentation of cancers when they used a deep learning model. Hering, A., et al. [70] reported a pipeline that successfully segmented and measured volumetric changes in soft tissue lesions in 96% of BL cases and 80% of FU images. The model was trained according to the RECIST criteria and can be an efficient and automated method of evaluating treatment response in FU images. Lee, C.-c., et al. and Patriarche, J. W. and B. J. Erickson [28, 71] similarly used deep learning models to compute volumetric changes based on intensity differences and evaluate treatment response in brain tumors after using the registration to align the images. The advantage observed in these deep learning models is that they can highlight relatively subtle changes while suppressing noise and normalization problems. They also do not require extensive user input which, in practice, can reduce the amount of data clinicians must review [28].

Six studies [58, 66, 69, 75–77] used deformation-based methods to quantity changes. Shearkhani, O., et al. [76] non-rigidly registered 50 pairs of T1-weighted MR images of metastatic brain tumor patients and analyzed the deformation fields to detect and quantify volumetric changes in the tumors. The Jacobian operator field (JOF), retrieved from the transformation matrix of the deformable registration, was used to compute the changes with a sensitivity of 85.1% for 1.5 T images and 92.1% for 3 T images. The algorithm’s ability to detect volume-changing tumors suggests its potential role in improving radiologist performance as a detection tool. Sakamoto, R., et al. [66] combined the deformation analysis with the subtraction method to study evolving nodules in CT images of the lung. Subtraction images visualized new or vanished nodules whereas the Jacobian maps presented growing or shrinking nodules. Tan, M., et al. [58] similarly analyzed transformation maps from deformable registration to compute volumetric changes of metastatic tumors in ovarian cancer patients undergoing drug treatment. The group showed that this method had a higher accuracy in predicting clinical outcomes than the traditional RECIST, suggesting incorporating volumetric analysis into RECIST may improve future diagnostic accuracy. Vandewinckele, L., et al. [69] reported a method that combined deformable registration with deep learning to segment five head and neck organs of patients undergoing radiotherapy. Although the model was trained on limited data, the model showed improvement in segmentation performance as shown by the Dice coefficient, and saved time compared to traditional methods such as large deformation diffeomorphic metric mapping (refer to Table 2 for a summary of the results).

**Table 2:** Summarizes the results of the articles related to the clinical application of registration-based change tracking in tumors including the type of registration and the method used to detect changes. We selected 5 examples of each method to detect changes

First author	Year	Type of registration	Change tracking method	Pathology	Summary of results
Choi, B., et al	2002	Nonrigid	Subtraction images	Breast lesions	The study reported a subtraction method and suggested that it was adequate for screening breast cancer lesions in dynamic contrast-enhanced MR mammography (CE MRM)
Abe, H., et al	2004	Both rigid and nonrigid	Subtraction images	Lung cancer	The reported method can significantly improve the sensitivity and specificity for the detection of early primary lung cancer on CT scans
Nakaura, T., et al	2008	Rigid	Subtraction images	Hepatocellular carcinoma	The suggested method can improve the performance of radiologists in detecting focal hepatic carcinoma lesions during the hepatic arterial phase of CT scans
Sakamoto, R., et al	2017	Nonrigid	Subtraction images	Bone metastases	The reported method significantly reduced the reading time of CT bone images and showed promising results in efficiently detecting new bone metastases
Khalil, A., et al	2021	Both rigid and nonrigid	Subtraction images	Brain tumor	The study showed that the subtraction method can help clinicians achieve early diagnosis of brain tumors on MRI and enhance management plans for patients
Yang, X., et al	2014	Nonrigid	Automatic segmentation and volumetric analysis	Head and neck tumor	The reported method of a novel automatic MR parotid gland segmentation algorithm based on atlas registration and machine learning can help address xerostomia in patients after head and neck radiation therapy
Fehr, D., et al	2016	Both rigid and nonrigid	Automatic segmentation and volumetric analysis	Metastatic bone tumors	The reported method of fully automatic detection and longitudinal tracking of bone metastasis lesions showed reasonable agreement with radiologists
Vandewinckel, L., et al	2020	Nonrigid	Automatic segmentation and volumetric analysis	Head and neck tumor	The reported method of automated segmentation of longitudinal data combined deformable image registration (DIR) and convolutional neural networks (CNN) and showed a significant improvement in automatically segmenting four head and neck organs in CT scans
Lee, C.-c., et al	2021	Rigid	Automatic segmentation and volumetric analysis	Vestibular schwannoma	The reported method of automating volumetric measurements of vestibular schwannoma on a series of parametric MR images after radiosurgery can be used in longitudinal follow-up assessments
Hsu, D. G., et al	2023	Rigid	Automatic segmentation and volumetric analysis	Metastatic brain tumors	The study reported an automatic longitudinal tracking method of brain metastases using deep learning methods. The accuracy in detecting and tracking tumor volumes seems adequate to support clinical workflows, except in tumors smaller than 1 cm^3^
Konukoglu, E., et al	2008	Both rigid and nonrigid	Deformation analysis	Meningioma	The reported method successfully measured the volumetric changes of slowly evolving meningioma in brain MRIs and highly correlated with expert findings
Chitphakdithai, N., et al	2012	Both rigid and nonrigid	Deformation analysis	Brain tumor	The reported method showed a high sensitivity for brain metastasis detection and volume estimation. The method can register images even with many months between scans
Tan, M., et al	2015	Nonrigid	Deformation analysis	Ovarian cancer	The reported method of CT images in this study yielded higher prediction accuracy of treatment response of ovarian cancer compared to RECIST guidelines
Shearkhani, O., et al	2017	Nonrigid	Deformation analysis	Metastatic brain tumors	The reported method can complement the performance of radiologists in detecting changes in metastatic brain tumors on MRIs with high accuracy
Sakamoto, R., et al	2014	Both rigid and nonrigid	Both subtraction and deformation analysis	Lung cancer	The reported method provided accurate registration of serial lung CT images and temporal subtraction images with Jacobian maps and can help radiologists find changes in pulmonary nodules

## Conclusions and Discussions

This review looked at how registration can help track changes in longitudinal images. We also examined the various automated methods of detecting and computing these changes and their application in MS and tumors. We focused on the clinical applications of MS and tumors, as these are two of the most extensively studied areas in automated change tracking. In terms of registration techniques, we explored the role of rigid or affine registration in 16 articles on MS and 5 on tumors. Nonrigid registration was reported in 10 articles on MS and 16 on tumors. A significant finding was observed in many articles was that nonrigid registration was often preceded by rigid and/or affine registration to minimize error and enhance performance. After pairs of longitudinal images are registered, various features (e.g., cysts, tumors, lesions) can be compared using different approaches. We found the intensity-based methods to be the most common methods for utilizing information from registration to detect and quantify changes. Consequently, we mainly reviewed articles employing these methods (23 articles on MS and 14 on tumors), with image subtraction being the predominant technique. Out of these intensity-based methods, Subtraction techniques were employed in most articles in MS and many articles in tumors and was mostly preceded by rigid or affine registration. The main clinical advantage of subtraction images is that they serve as a reference for clinicians to enhance their performance and improve the accuracy of lesion detection. However, subtraction methods have limitations such as susceptibility to noise and restriction to voxel-wise changes. Therefore, we examined some studies that further improved intensity-based change detection methods with probabilistic classification algorithms, which incorporate contextual information to better differentiate between true changes and artifacts. However, these algorithms require complex modeling and may need large training datasets.

Furthermore, one of the most promising methods we observed was the deep learning-based methods for registration and change tracking. Compared to traditional methods, deep learning can provide automated functions that can increase accuracy, save time, and reduce user bias. These automated methods can be significantly impactful by providing clinicians with a computer-aided diagnostic tool. Several studies in this review employed deep learning and automated methods for image subtraction and segmentation, demonstrating high sensitivity and reduced false positives (please refer to Table 1 and Table 2 to find multiple examples and studies). However, deep learning methods have many limitations that should be acknowledged as well. Particularly, the need for large annotated datasets which can be time consuming, and its performance can vary based on the dataset. Additionally, they cannot yet capture every type of change, especially in diseases like polycystic kidney disease where the disease phenotype is complex. For instance, kidney cysts can change in size, number, shape, and composition (when cysts become infected or hemorrhagic) and predicting all these changes with the model would be a difficult task.

Therefore, future research should focus on increasing generalizability and robustness so that these models can be applied accurately to every type of disease presentation with great accuracy. This can be achieved by training and validating models on multi-institutional datasets that represent diverse patient populations, diseases, and image modalities [9]. Additionally, one of the critical advantages of deep learning-based algorithms is that after they have been trained, they can then be applied to perform registration non-iteratively and rapidly without the need for complex optimization computations for each case. Unlike traditional methods, they also require minimal pre-processing of the images and minimize user bias. Thus, another important area that future research can focus on is developing more advanced deep learning models that can perform registration non-iteratively and more rapidly [16].

The scope of this review contained only MS and tumors, and it is worth mentioning that multiple articles in the literature explored other clinical applications. For instance, imaging of the retina is one important area in which the role of registration has been investigated. The systematic review by Pan, L. and X. Chen [78] reviewed registration methods in retina OCT images and reported that the use of deep learning in retinal registration is currently limited to 2D images. However, there is now ongoing research on using DL technology to obtain 3D information to solve more complex problems in OCT images. Other areas include the role of registration in diseases such as epilepsy. Chitphakdithai, N. and J. S. Duncan [79] described an intensity-based method that improved image alignment compared to a traditional registration algorithm by handling missing correspondence of the resection region in the brain after surgery for epilepsy. Articles about registering images to help track changes in pulmonary and bone diseases are other examples of applications beyond the scope of this review.

Another limitation is covering registration and change tracking in the context of only longitudinal images. Registration also plays a critical role in real-time image tracking, particularly in radiation oncology. Its role has been studied in many aspects of radiotherapy ranging from image-guided and adaptive radiotherapy to motion management, and response evaluation. We focused exclusively on longitudinal images to provide a comprehensive analysis of the role of registration in this specific area. Because we noticed that multiple reviews have addressed the real-time aspect, but there is a notable scarcity of literature focusing on longitudinal images. It also worth mentioning that the use of artificial intelligence is being explored not only the longitudinal images, but also in the real-time image registration, and could potentially be the method of choice in the future [9]. However, it is outside the scope of our study.

In conclusion, the literature contains a limited number of articles on the role of registration in longitudinal images, and we believe this systematic review offers a comprehensive overview of the role of registration-based approaches in change tracking in radiological imaging as well as main methods of detecting and quantifying changes in registered longitudinal images.

## Data Availability

The list of articles used in this review are available from the corresponding author upon request.
